# Bacterial contaminants of stored blood and blood components ready for transfusion at blood banks in Mekelle, Northern Ethiopia

**DOI:** 10.1186/s13104-019-4217-0

**Published:** 2019-03-25

**Authors:** Masresha Agzie, Selam Niguse, Ephrem Tsegay, Getahun Kahsay, Mahmud Abdulkader Mahmud

**Affiliations:** 1 North Command Blood Bank, Mekelle, Ethiopia; 2 Department of Medical Microbiology and Immunology, College of Health Science, Mekelle University, P. O. Box 1871, Mekelle, Ethiopia

**Keywords:** Stored blood, Bacterial contamination, Antimicrobial susceptibility, Mekelle, Blood bank

## Abstract

**Objective:**

Bacterial contamination of donated blood and blood components is a major public health problem globally. The aim of the study was to evaluate the rate and spectrum of bacterial contaminations and antimicrobial susceptibility pattern of contaminants in stored blood and blood components.

**Results:**

A total of 196 blood and blood components (concentrated red blood cells, fresh frozen plasma, and platelets) were included. Bacterial contamination was observed in 18 (9.2%) of the blood and blood components, of which 14 (77.8%) and 4 (22.2%) were gram positive and gram negative bacteria, respectively. The predominantly isolated bacteria were Coagulase-negative Staphylococcus, Bacillus spp., and *Staphylococcus aureus*. Majority of isolated gram-negative bacteria isolates showed resistance to tetracycline and doxycycline. Multidrug resistance was observed in 12 (66%) of the isolates.

## Introduction

Bacterial contamination of donated blood is defined as the presence of bacteria in the blood or blood components which are collected and/or processed for transfusion [1]. A ready to be transfused blood should be free from microbial contaminants including bacteria [2]. For this blood should be collected and processed following aseptic technique [3]. However, bacterial contamination of donated blood may occur as a result of endogenous (from the donor) or exogenous (during collection and processing) route [4, 5].

Bacterial contamination of donated blood can be observed in different mechanisms [6]. The survival of bacteria in red blood cell may produce gas, resulting in unnecessary and unusual air bubbles. This results in a pink to red discoloration that could be seen in the supernatant [7]. Bacterial contamination of donated blood (whole blood, concentrated red blood cell, and platelets) have been observed to cause a severe problem in transfusion therapy in the previous 20 years and is the second only to ABO-mismatch in causing transfusion-associated death [8].

Globally, the exact prevalence of bacterial contamination of blood and blood Components is unknown [9]. However several studies showed that bacterial contamination of donated blood was, 0.2%, 0.15%, and 0.1% in the United States of America, UK, and France, respectively [10–13]. It was indicated in various studies that the factors that promote bacterial contamination of donated blood include; touching disinfected phlebotomy site, in proper use disinfection, and double puncture at the same hand or both hands of a donor, improper storage of blood and donor bacteremia [13].

Other studies from sub-Saharan African countries showed that the prevalence of contamination of donated blood is higher than that of developed countries [14–19]. Except few studies done in Gonder and Debre Markos [20, 21], bacterial contamination of donated blood in Ethiopia has not been given due attention unlike that of transfusion-transmitted viral infection. The above studies done in Ethiopia showed considerable and variable prevalence of bacterial contaminations among blood donors were reported. This calls for further research to be done in different settings. Therefore, this study will evaluate the rate of bacterial contamination of stored blood and blood components at North Command Army and Tigray Region Blood Banks.

## Main text

### Methods

A cross-sectional study was conducted from February to April 2017 in North Command Army and Tigray Region Blood Banks. The blood banks are situated in Mekelle, Northern Ethiopia. The sample size was calculated using a single population proportion formula, considering, 95% confidence interval, a margin of error, d = 0.05 and previous prevalence of bacterial contamination on stored blood, p = 15% [19]. A total of 196 stored blood bags were selected for the study. Collection of blood donations in the respective blood banks was according to the standard operating procedures (SOPs) and the average annual blood donations were 1800 and 9000 blood bag units in North Command Army and in Tigray Regional Blood Banks, respectively. The sample size was proportionally allocated to the two blood banks. Accordingly, 163 and 33 stored blood bag units were selected using a systematic sampling technique from North Command Army and Tigray Regional Blood Bank, respectively. Sample processing, transportation, and analysis were done using standard bacteriological safety and aseptic techniques. In this study, both stored blood and blood products bag units (including concentrated red blood cells, fresh frozen plasma, and platelets) were included. Stored blood and blood product bags were thoroughly mixed, and the end of the tied tubing was swabbed, disinfected, and cut with sterile scissors. Some of the mixed blood from the main bag was allowed to seep into the line. The end of each line was clipped with sterile forceps to prevent blood from flowing back into the main bag. These cut ends were directly transported to Ayder Comprehensive Specialized Hospital laboratory Microbiology department for sample processing and laboratory analysis, following protocols stated elsewhere [22]. Two knots were made on the line, and the last knot was swabbed with 70% ethanol and punctured with a sterile needle and syringe to draw 5 mL of blood product. The samples were dispensed into separate, sterile bottles containing 50 mL of Brain–Heart Infusion (BHI) broth in culture bottle. All of the suspensions were incubated aerobically at 37 °C for up to seven days and observed for signs of bacterial growth on days 2 and 7. For samples showing signs of bacterial growth, a gram smear was made and examined microscopically. At the same time, the samples were subcultured using standard methods onto Blood agar (BA), chocolate agar (CA), and MacConkey agar (MA). Blood agar and MA plates were incubated aerobically, while CA plates were incubated in candle jars at 37 °C for up to 48 h. Plates were inspected for bacterial growth at 24 h (BA, CA, and MA) and 48 h (BA and CA). Further microbiological identification of isolates was done using standard biochemical tests. Antimicrobial susceptibility test was performed using the modified Kirby–Bauer disk diffusion method according to the Clinical Laboratory Standard Institute guidelines [23]. Sterile swabs were used to seed colonies into Muller Hinton Agar to give a confluent bacterial growth. Using a pair of sterile forceps the antimicrobial disks for gram-positive isolates; chloramphenicol (30 µg), penicillin (10 unit), erythromycin (15 µg), cefoxitin (30 µg) and clindamycin (2 µg), or gram-negative isolates; tetracycline (30 µg), amikacin (30 µg) and Augmentin (10 µg),common drug for gram positive and gram negative; cefotaxime (30 µg), tobramycin (10 µg), doxycycline (30 µg), ampicillin (10 µg), gentamicin (10 µg), ciprofloxacin (5 µg) and augmenting (10 µg) were placed on the dry agar surface as appropriate. Then the plates were incubated aerobically at 37 ºC and read after 18–24 h of incubation. Zones of inhibition surrounding the disks were measured using a caliper. The organisms were reported as sensitive, intermediate or resistant. Known reference strains of *Pseudomonas aeruginosa* (ATCC 27853), *E. coli* (ATCC 25922) and *S. aureus* (ATCC 25923) were used for quality assurance [24]. Data were analyzed by Statistical Package for Social Sciences (SPSS) software version 22.0. (IBM, USA). Descriptive statistics were computed and data were presented using tables.

## Results

In this study, a total of 196 blood and blood components were included. Of which 163 (83.2%) and 33 (16.8%) were from Tigray regional blood bank and North command Army blood bank, respectively. During the time of sample collection majority of the samples were whole blood, 143 (72.9%) followed by concentrated red cell, 27 (13.8%) and fresh frozen plasma, 14 (7.1%) and platelets, 12 (6.1%). Of the blood collected, 63 (32.1%) were stored for 7–8 days.

In this study, out of 196, 18 (9.2%) of the blood and blood components were found contaminated with bacteria. From Tigray Region Blood Bank, 14 (8.5%) and from North Command Army Blood Bank, 4 (1.2%) of the blood components were contaminated with bacteria. Bacterial contamination of whole blood was 9 (6.3%) followed by concentrated red cell 5 (4.6%). From the total 18 isolates, 14 (77.8%) and 4 (22.2%) were gram positive and gram negative bacteria, respectively. The predominantly isolated bacteria were Coagulase negative Staphylococcus 5 (27.7%), followed by *Bacillus* spp.*,* 4 (22.2%), *Staphylococcus aureus* 3 (16.6%), *Pseudomonas aeruginosa* 3 (16.6%), *Streptococcus pneumoniae* 2 (11.1%) and *Escherichia coli* 1(5.5%). The majority, *Coagulase-negative Staphylococcus *spp.,* Bacillus *spp.*, Staphylococcus aureus, Streptococcus pneumoniae* were isolated in the 0–3 day stored blood and blood components (Table 1).

**Table 1:** Storage time and bacterial contamination of stored blood and blood products at blood bank

Time of storage (days)	Bacteria isolated	Frequency, N
0	*Streptococcus pneumoniae*	2
7	*Pseudomonas aeruginosa*	1
8	*Pseudomonas aeruginosa*	1
3	*Escherichia coli*	1
3	*Pseudomonas aeruginosa*	1
3	*Bacillus *spp.	1
6	*Bacillus *spp.	1
2	*Staphylococcus aureus*	1
5	*Coagulase negative Staphylococcus*	1
2	*Coagulase negative Staphylococcus*	3
1	*Coagulase negative Staphylococcus*	1
5	*Staphylococcus aureus*	1
2	*Bacillus *spp.	1
4	*Bacillus *spp.	1
1	*Staphylococcus aureus*	1

Among gram positives *Streptococcus pneumonia, Staphylococcus aureus*, *Bacillus* spp. and Coagulase negative Staphylococcus of the isolates was sensitive to chloramphenicol, 13 (93%), clindamycin, 13 (93%), ciprofloxacin, 9 (64%), gentamicin, 8 (57%) doxycycline 5 (36%), cefoxitin, and Tobramycin 1(7%). *Staphylococcus aureus* showed 3 (100%) resistance to cefotaxime, Penicillin, and erythromycin (Table 2). Majority of isolated Gram-negative bacteria isolates showed increased resistance to tetracycline and doxycycline. However, all gram-negative isolates showed 100% sensitivity to both ciprofloxacin and gentamycin. *Pseudomonas aeruginosa* showed a high level of resistance to tetracycline and doxycycline. In addition to this, increased resistance of *Escherichia coli* to ampicillin, tetracycline, and amikacin was also observed (Table 3). Multidrug resistance was seen in 12 (66%) of the isolated bacteria.

**Table 2:** Antimicrobial susceptibility pattern of Gram-positive bacteria isolated from blood and blood component

Isolates (N)	Pattern	CXT	AMP	PEN	ERY	CAF	CLI	CIP	DOX	CEF	GEN	TOB
*S.pneumoniae* (2)	S	0 (0)	0 (0)	0 (0)	0 (0)	2 (100)	2 (100)	NA	NA	0 (0)	NA	NA
	I	0 (0)	0 (0)	0 (0)	0 (0)	0 (0)	0 (0)	NA	NA	0 (0)	NA	NA
	R	2 (100)	2 (100)	2 (100)	2 (100)	0 (0)	0 (0)	NA	NA	2 (100)	NA	NA
*S.aureus* (3)	S	0 (0)	NA	0 (0)	0 (0)	3 (100)	3 (100)	3 (100)	2 (67)	NA	3 (100)	NA
	I	0 (0)	NA	0 (0)	0 (0)	0 (0)	0 (0)	0 (0)	0 (0)	NA	0 (0)	NA
	R	3 (100)	NA	3 (100)	3 (100)	0 (0)	0 (0)	0 (0)	1 (33)	NA	0 (0)	NA
*CoNS* (5)	S	5 (100)	NA	0 (0)	1 (20)	4 (80)	4 (80)	3 (60)	3 (60)	1 (20)	5 (100)	NA
	I	0 (0)	NA	0 (0)	1 (20)	1 (20)	1 (20)	2 (40)	2 (40)	2 (40)	0 (0)	NA
	R	0 (0)	NA	5 (100)	3 (60)	0 (0)	0 (0)	0 (0)	0 (0)	2 (40)	0 (0)	NA
*Bacillus Spp*. (4)	S	0 (0)	NA	2 (50)	2 (50)	4 (100)	4 (100)	3 (75)	NA	NA	3 (75)	1 (25)
	I	1 (25)	NA	0 (0)	0 (0)	0 (0)	0 (0)	0 (0)	NA	NA	1 (25)	2 (50)
	R	3 (75)	NA	2 (50)	2 (50)	0 (0)	0 (0)	1 (25)	NA	NA	0 (0)	1 (25)
Total (14)	S	5 (36)	0 (0)	2 (14)	3 (21)	13 (93)	13 (93)	9 (64)	5 (36)	1 (7)	11 (78)	1 (7)
	I	1 (7)	0 (0)	0 (0)	1 (7)	1 (7)	1 (7)	2 (14)	2 (14)	2 (14)	1 (7)	2 (14)
	R	8 (57)	2 (14)	12 (86)	10 (71)	0 (0)	0 (0)	1 (7)	1 (7)	4 (29)	0 (0)	1 (7)

*CXT* Cefotaxime, *AMP* ampicillin, *PEN *penicillin, *ERY* erythromycin, *CAF* chloramphenicol, *CLI* clindamycin, *CIP* ciproflaxin, *DOX* doxycycline, *CEF* cefoxitin, *TOB* tobramycin, *GEN* gentamicine, *NA* not applicable

**Table 3:** Antimicrobial susceptibility pattern of Gram-negative bacteria isolated from blood and blood component

Isolates (n)	Pattern	TOB	AMP	CXT	TTC	AMK	DOX	CIP	GEN	AUG
*P. aeruginosa* (3*)*	S	0 (0)	NA	1 (25)	0 (0)	0 (0)	0 (0)	3 (100)	3 (100)	NA
	I	2 (67)	NA	0 (0)	0 (0)	2 (67)	0 (0)	0 (0)	0 (0)	NA
	R	1 (33)	NA	2 (75)	3 (100)	1 (33)	3 (100)	0 (0)	0 (0)	NA
*E. coli* (1)	S	1	0 (0)	1	0 (0)	0 (0)	1	1	1	1
	I	0 (0)	0 (0)	0 (0)	0 (0)	0 (0)	0 (0)	0 (0)	0 (0)	0 (0)
	R	0 (0)	1	0 (0)	1	1	0 (0)	0 (0)	0 (0)	0 (0)
Total (4)	S	1 (25)	0 (0)	2 (50)	0 (0)	0 (0)	1 (25)	4 (100)	4 (100)	1 (25)
	I	2 (50)	0 (0)	0 (0)	0 (0)	2 (50)	0 (0)	0 (0)	0 (0)	0 (0)
	R	1 (25)	1 (25)	2 (50)	4 (100)	2 (50)	3 (75)	0 (0)	0 (0)	0 (0)

*TOB* Tobramycin, *AMP* ampicillin, *CXT* cefotaxime, *TTC* tetracycline, *AMK* amikacine, *DOX* doxycycline, *CIP* ciproflaxin, *GEN* gentamicine, *AUG* augumentine, *NA* not applicable

### Discussions

Though blood banks have standard operating procedures to minimize bacterial contamination of donated and stored blood bags, there are reports of bacterial contamination from different blood banks with different rates. Performing studies focusing on bacterial contamination of stored blood is important to provide information to policymakers on the safety of the collected blood. This is a cross-sectional study focusing on evaluating the rate of bacterial contamination of stored blood and blood products. In the study, the prevalence of bacterial contamination of blood and blood component was 9.2%. The bacterial contamination on this study is lower than other similar studies done in Gondar and Debre Markos [19, 20], Ghana; 17.5% [17] and Egypt 17.9% [25]. However, result of this study is relatively higher than study conducted in America; 0.2% [10], UK; 0.15%, [11], France; 0.1 [12], Zimbabwe; 3.1% [26], Uganda; 3.5% [27]. The difference for higher prevalence in this study is might be due to poor infrastructure setup and practice of infection prevention standards, recent achievements of the screening test for viral contamination of donor’s blood, have obscured the need of screening for bacterial contaminates [28].

Unlike most of the study reports in our country and elsewhere in the world, our findings showed that majority of the etiological agents for bacterial contamination of stored blood were gram-positive bacteria. In this study, *Coagulase-negative Staphylococcus* was the most predominant isolate with an isolation rate of 5 (27.7%), which is in line with studies conducted in the same country; (Gonder and Debre Markos) [19, 20]. and in most of the studies conducted in African countries, such as Kenya [13]. Nigeria [14], and Ghana [17]. The major contributing factor for isolating such higher rate CoNS might be due to the improper disinfection procedures during blood donor collection. The fact that *Bacillus *spp. was the second dominant bacterial isolates, in this study, might be related to poor skin cleansing techniques before donor blood is obtained. Comparable results were reported from other previous studies conducted in Kenya [13]. On top of this finding of *Pseudomonas aeruginosa* in this study was also supported by other studies from Kenya and Ghana [13, 17]. A high level of bacterial contamination was observed within 0–3 days of storage. Similar results were observed in different studies. A study from Denmark on fresh donated blood high level of bacterial contamination 21 (35%) of 60 RBC-fractions was reported [29]. Moreover, other studies in Kenya, Ethiopia, Ghana and Nigeria significant level of bacterial contamination reported within short period of storage time [13, 14, 17, 20]. In this study, a high level of bacterial contamination was observed in the platelets and concentrated red cell. The possible explanation for the higher bacterial contamination of blood components more susceptibility for bacterial contamination during component processing and agitation. Platelets are stored between 22 °C and 24 °C with constant agitation, which is favorable for bacterial proliferation [26, 27].

Considering the drug resistance pattern, the finding s of this study were similar other similar studies done in Ethiopia [19, 20]. According to the international standard for the definition of drug resistance [30], Multidrug resistance (MDR = Non-susceptible to ≥ 1 agent in ≥ 3 antimicrobial categories) was observed in12 (66.6%). Of the total isolated bacterial contamination of stored blood. This was in line with the finding of a study conducted in Gondar Hospital Blood Bank North West Ethiopia [19]. But it is higher than that of Debre Markose Referral Hospital Northwest Ethiopia [20] and Mbarara Regional Blood Bank South Western Uganda. On the other hand, it is lower than that of Tertiary Hospital Nigeria [14].

In conclusion, bacterial contamination was observed in 18 (9.2%) of the blood and blood components, of which 14 (77.8%) and 4 (22.2%) were gram positive and gram negative bacteria, respectively. In addition high resistance patterns were observed for a single and multiple antimicrobials that need urgent attention. Therefore, blood bank centers should improve their standard in infection prevention of bacterial contamination of donated blood. Further study should be conducted to determine the sources of bacterial contamination of stored blood and its components.

## Limitations

The small sample size in the blood components was difficult to make a descriptive analysis about the blood components and further analyze the factors associated with bacterial contamination.

## Abbreviations

BHI: Brain–Heart Infusion
CA: chocolate agar
CoNS: Coagulase negative Staphylococcus
CRC: concentrates red cell
MAC: MacConkey
TTI: transfusion transmitted infections
WHA: World Health Assembly
WHO: World Health Organization

## References

[1] Acker J, Andres R. reference guide, visual inspection donated blood. American red cross biomedical services, fraser health authority, british columbia. 2009;T05(021):9–10.

[2] Walther G (2008). Incidence of bacterial transmission and transfusion reactions by blood components. Clin Chem Lab Med..

[3] Goodrich R, Gilmour D, Hovenga N (2009). Laboratory comparison of pathogen reduction technology treatment and culture of platelet products for addressing bacterial contamination concerns. Transfusion.

[4] Cawley C, McDonald C, Ancliff S (2011). Early recognition and reporting of suspected bacterial contamination may prevent transfusion transmission of infection by associated units. Transfus Med.

[5] Arewa O (2009). One year clinical audit of the use of blood and blood components at a tertiary hospital in Nigeria. Niger J Clin Pract.

[6] Schmidt M, Sireis W, Seifried E (2011). Implementation of bacterial detection methods into blood donor screening—overview of different technologies. Transfus Med Hemother.

[7] Müller T, Montag T, Seltsam A (2011). Laboratory evaluation of the effectiveness of pathogen reduction procedures for bacteria. Transfus Med Hemother.

[8] Otsubo H, Yamaguchi K (2008). Current risks in blood transfusion in Japan. Jpn J Infect Dis.

[9] Perkins H, Busch M (2010). Transfusion-associated infections: 50 years of relentless challenges and remarkable progress. Transfusion.

[10] Kuehnert V, Roth N (2001). Transfusion transmitted bacterial infection in the United States, 1998 through 2000. Transfusion.

[11] Williamson LM, Lowe S, Love EM, Cohen H, Soldan K, McClelland DBL (1999). Serious hazards of transfusion (SHOT) initiative: analysis of the first two annual reports. BMJ.

[12] Wanger SJ, Friedman LI, Dodd RY (1994). Transfusion-associated bacterial sepsis. Clin Microbiol Rev..

[13] Hassall O, Maitland K, Pole L (2009). Bacterial contamination of pediatric whole blood transfusions in a Kenyan hospital. Transfusion.

[14] Aboderin O (2011). Bacterial contamination of blood and blood components in a tertiary hospital setting in Nigeria. Int J Infect Control.

[15] Opoku C, Feglo P (2009). Bacterial contamination of donor blood at the Tamale Teaching Hospital Ghana. Afr Health Sci.

[16] Boye A, Samuel A, Daniel D (2016). Bacterial contamination of at-point-of transfusion blood in a tertiary hospital in Ghana. EC Bacteriol Virol.

[17] Adjei A, Kuma G, Tettey Y (2009). Bacterial contamination of blood and blood components in three major blood transfusion centers, Accra Ghana. Jpn J Infect Dis.

[18] Samia A, Ghada A (2014). Rapid detection of bacterial contamination in platelet concentrates, by polymerase chain reaction and DNA sequencing in comparison to conventional automated culture. J Curr Microbiol App Sci.

[19] Wondimu H, Addis Z, Moges F (2013). Bacteriological safety of blood collected for transfusion at university of Gondar hospital blood bank, northwest Ethiopia. ISRN Hematol.

[20] Ahmed E, Zewdu D (2014). Bacterial contamination of donated blood ready for transfusion at a Referral Hospital in Ethiopia. J Clin Res Bioeth.

[21] Chelsea A, Sheppard M, Cassandra D (2005). Review on bacterial contamination of platelets for transfusion: recent advances and issues. Lab Med.

[22] Victor W, Daniel M, William M. Guide to laboratory services: microbiology. Arizona Department of health service Bureaus of state laboratory service, 01. 20. 2017.

[23] Clinical and Laboratory Standards Institute (CLSI). Performance standards for antimicrobial susceptibility testing. Clinical and Laboratory Standards Institute: Wayne, 2017, p. M100–S25.

[24] Centers for disease control and Prevention (CDC). Biosafety in microbiological and biomedical laboratories USA: 2009. HHS Publication No.(CDC) 21-1112.

[25] Samia A, Ghada A, Fatama H (2014). rapid detection of bacterial contamination in platelet concentrates by polymerase chain reaction and DAN sequencing in comparison to conventional automated culture. Int J Curr Microbiol Appl Sci.

[26] Makuni N (2015). Prevalence of bacterial contamination in blood and blood components at the National Blood Service Zimbabwe. J Infect Dev Ctries.

[27] Aloysius G (2013). Bacterial contamination of blood and blood components at Mbarara Regional Blood Bank in Rural South Western Uganda. Adv Infect Dis.

[28] Alex K, Owusu O (2012). Transfusion-transmitted malaria and bacterial infections in a malaria endemic region.

[29] Damgaard C, Magnussen K, Enevold C, Nilsson M, Tolker-Nielsen T, Holmstrup P (2015). Viable bacteria associated with red blood cells and plasma in freshly drawn blood donations. PLoS ONE.

[30] Magiorakos AP, Srinivasan A, Carey RB, Carmeli Y, Falagas ME, Giske CG (2012). Multidrug-resistant, extensively drug-resistant and pan drug-resistant bacteria: an international expert proposal for interim standard definitions for acquired resistance. Clin Microbiol Infect.

